# Short-term application of ibuprofen before ovulation

**Published:** 2020-10-08

**Authors:** AS Kohl Schwartz, S Burkard, VR Mitter, AB Leichtle, A Fink, M Von Wolff

**Affiliations:** University Women’s Hospital, Division of Gynecological Endocrinology and Reproductive Medicine, Inselspital, Bern University Hospital, Bern, Switzerland; Department of Clinical Chemistry, Inselspital, Bern University Hospital, Bern, Switzerland

**Keywords:** Iboprufen, implantation rate, IVF, natural cycle IVF, non-steroidal anti-inflammatory drug, ovulation

## Abstract

**Aim of the study:**

The aim was to analyse if ibuprofen, as a non-selective cyclooxygenase (COX) inhibitor, has any negative effect on oocyte competence and embryo quality. COX- inhibitors are popular over-the-counter analgesics. Whereas selective COX inhibitors have been shown to impair female fertility, data on non-selective COX inhibitors are poor. Hence, they have not been recommended for women trying to conceive.

**Methods:**

This is an observational study comparing ibuprofen exposed and unexposed women from 18 to 42 years of age, using the model of natural cycle in vitro fertilisation (IVF) to determine oocyte and embryo quality. Follicular growth was monitored and if the follicle was mature (≥ 15mm size and estimated oestradiol level of ≥ 800pmol/l), ovulation was triggered. Women with luteinising hormone (LH) surge received 400mg ibuprofen every 8 hours to postpone ovulation, whereas women without LH surge received none (controls). Oocyte retrieval rate, oocyte maturity, fertilization rate, embryo development and embryo quality as well as implantation rate were analysed.

**Results:**

Of the 111 women included, 63 received ibuprofen, and 48 did not. Rates of mature oocytes and implantation rate did not differ. Logistic regression showed no significant association of ibuprofen intake, LH- level or reason for infertility on embryo quality.

**Conclusion:**

Based on our results, we suggest that, particularly within natural cycle IVF, ibuprofen does no harm around ovulation as analgesic treatment.

## Introduction

Non-selective cyclooxygenase (COX) inhibitors, such as ibuprofen, are over-the-counter analgesics frequently used by reproductive-aged women. They are highly effective in the treatment of pain conditions, such as dysmenorrhoea, without greatly affecting the gastrointestinal tract, when using standard dosages (Kellstein et al., 1999). Cyclooxygenase is one of the key enzymes involved in ovulation and implantation (Duffy, 2015). Ovulation is a complex process induced by the luteinizing hormone (LH) surge. The pre-ovulatory LH surge stimulates the expression of COX II and thereby increases the production of prostaglandins by granulosa cells, especially prostaglandin E2 (PGE2) (Ricciotti and Fitzgerald, 2011). PGE2 stimulates and modulates the production of follicular matrix metalloproteases (MMP), which degrade parts of the cumulus complex surrounding the oocyte, which then detaches from the follicular wall. It also induces lysis of the follicular wall, leading to the release of the follicular fluid containing the detached oocyte into the fallopian tube (O’Sullivan et al., 1997).

Because all these processes are initiated, regulated or modulated by prostaglandins, COX inhibitors could have a substantial impact on ovulation and possibly on oocytes.

Indeed, selective COX inhibitors such as rofecoxib and meloxicam have been shown to substantially inhibit ovulation (Pall et al., 2001). Furthermore, it has been shown that oocytes from very large follicles, e.g. after prolonged stimulation, have a lower implantation potential (Ectors et al., 1997; Wirleitner et al., 2018). Accordingly, the inhibition of ovulation with non-selective COX inhibitors might prolong follicular maturation, negatively affect oocyte quality, and thereby reduce pregnancy rates.

However, only limited data is available on the impact of non-selective COX inhibitors on oocyte quality and pregnancy rates. Some data is available on indomethacin (Kadoch et al., 2008) and diclofenac (Kawachiya et al., 2012), which postpone ovulation for a few hours without negative effects on oocyte maturation, fertilisation and pregnancy rates. The effect of ibuprofen, which is widely used in women due to its high efficacy in dysmenorrhea and its low impact on the gastrointestinal tract, (Kellstein et al., 1999; Bjarnason et al., 2018) has so far been only analysed by Uhler et al. (2001) who only studied its effect on ovulation but not on oocyte and embryo quality.

We therefore performed a study using the model of natural cycle in-vitro-fertilisation (NC-IVF) to investigate the impact of ibuprofen on oocyte quality and embryo quality by analysing primarily fertilization rate, oocyte quality, embryo quality and implantation rate.

## Methods

This is an observational cohort study. We retrospectively collected data comparing exposed to unexposed woman in unstimulated natural IVF cycles performed between 2014 and 2016 at the Berne University Hospital, Switzerland. All women included were 18 to 42 years of age with regular menstrual cycles, having chosen NC-IVF for their infertility treatment. NC-IVF served as a model for the natural menstrual cycle. Not receiving ovarian stimulation allows natural follicle recruitment and selection. Follicular growth was monitored once by transvaginal ultrasound, and if a follicle size of ≥15mm and oestradiol concentrations of ≥800pmol/l were expected, ovulation was induced by the injection of 5.000 IU of human chorionic gonadotrophin (hCG).

Ibuprofen is taken in NC-IVF to reduce the risk of premature ovulation in cases when the imminent LH surge begins (>10 IU/l) (Kawachiya et al., 2012). These women were triggered with hCG on the same day of follicular monitoring and received 400mg ibuprofen every eight hours until oocyte pick-up (OPU) two days later. In total 6 x 400mg were taken within 38-48 hours. Women with LH concentrations ≤10 IU/l did not receive ibuprofen (control group). We only included women with a known LH level at the specific day of ovulation triggering.

Directly before OPU an ultrasound was performed to measure the follicular size and to exclude premature ovulation. Follicle aspiration and follicular flushing (Von Wolff et al., 2013) was performed 36 hours after hCG injection using a single-lumen 19 Gauge needle. After successful oocyte collection (oocyte retrieval rate), oocyte maturity was evaluated according to the ESHRE consensus (Balaban et al., 2011) by a blinded embryologist (oocyte maturity rate). Metaphase II (MII) oocytes were fertilised by intracytoplasmic sperm injection, ICSI (fertilization rate). Fertilization rate was assessed 18h after ICSI. Embryos were cultured solely in global total (Life Global © ) and were monitored daily until transfer. Embryo development and quality before transfer was assessed using the ASEBIR embryo assessment criteria (Balaban et al., 2011). Embryo transfer of one embryo was performed at the cleavage stage (day 2-3 after ICSI) using soft uterine transfer catheters (Wallace ® , Smiths Medical, Kent, United Kingdom). For luteal phase support, following the choice of the patient, micronised progesterone (Crinone ® 8%, Merck, Darmstadt, Germany) or (Utrogestan ® , Vifor, Villars-sur-Glâne, Switzerland) was applied vaginally for at least 14 days. Serum HCG concentration was analysed 14-16 days after follicle aspiration. The HCG test was defined as positive (implantation rate) in case of β-HCG >30 IU/l. Delivery reports were collected from the referral clinics nine months later to assess live birth rates.

Statistical analysis was performed using STATA statistical software Version 14 (Stata Corporation, College Station, TX, USA). Normality was assessed by Q-Q plotting. Differences between groups were calculated using Student’s t-test for continuous variables and the Chi-Square test for categorical variables. The primary outcome criteria were embryo development rate and embryo quality to analyse the effect of ibuprofen of oocyte competence. The secondary outcome criteria were implantation and delivery rates. A p-value of < 0.05 was considered statistically significant. An ordered categorical regression model for embryo quality according to the ASEBIR embryo assessment criteria (A>B>C>D) as an outcome was performed, adjusting for the LH- level (continuous, in IU/l), the reason for infertility (male factor, tube factor, endometriosis and idiopathic infertility), the mother’sage (>35 years) and the ibuprofen exposure(binary).

Ethical approval was obtained from the Ethics Committee of Bern, Switzerland (KEK 397/15).

## Results

A total of 111 women were included in the study. There were no significant differences in the patient`s characteristics between the exposed (n=63) and the control (n=48) groups (Table I). The mean age was 35.4 (±4.3) years and the mean BMI was 22.3 (±3.4) kg/m2. The most frequent indication for reproductive treatment in both groups was male factor infertility (in total 61/111; 55%). The rate of women with a very low anti-mullerian hormone (AMH) concentration (defined as AMH <3.6pmol/l) was <20% in both groups. Most of the women were healthy (no chronic diseases) and did not use any regular co-medication.

**Table I:** Patient characteristics.

Characteristics	All women included (n = 111)	Ibuprofen exposed group (n= 63)	Control group (n = 48)	p-value
Age (years), mean ± SD	35.4 ± 4.3	35 ± 4.2	35.9 ± 4.3	0.271 ^1^
Body-Mass-Index(kg/m 2 ), mean ± SD	22.3 ± 3.4	22.7 ± 3.3	21.8 ± 3.6	0.220 ^1^
Aetiology of infertility, n (%)				0.614 ^2^
- Male Factor	61 (55)	38 (60.3)	23 (47.9)	
- Tubal Factor	2 (21.8)	2 (4.8)	0	
- Endometriosis	21 (18.9)	12 (19)	9 (18.8)	
- Idiopathic	27 (24.3)	11 (17.5)	16 (33.3)		
AMH in pmol/L, mean (range)	16.5 (0.1 - 87.1)	17.3 (0.1-87.1)	15.4 (1-76.4)	0.614 ^2^
- AMH <3.6 pmol/L	19 (17.1)	10 (15.9)	9 (18.8)	
Chronic Disease	16.5 (0.1 - 87.1)	17.3 (0.1-87.1)	15.4 (1-76.4)	0.805 ^2^
- None	82 (73.9)	56 (88.9)	34 (70.8)	
- Hypothyreosis (medically controlled)	4(3.6)	2(3.2)	2(4.2)	
- Depression / Eating disorder	5 (4.5)	1 (1.6)	4 (8.3	
- Diabetes	1 (0.9)	1 (1.6)	0	
- Cardiovascular disease	1 (0.9)	0	1 (2.1)	
- Asthma / neurodermitis	6 (5.4)	2 (3.2)	4 (8.3)	
- SLE/ juvenil rheumatiod Arthritis	4 (3.6)	1 (1.6)	3 (6.3)	
Follicles				0.727 ^1^
- Diameter in mm, mean (range)	18 (15 – 23)	18 (15 – 23)	19 (15 – 23)	
Oestradiol concentration on trigger day (pmol/L), mean (range)	1057.2 (177 – 2766)	1150.4 (540 – 2766)	934.8 (171 – 1680)	<0.005 ^1^
Luteinising hormone concentration on trigger day of (IU/L), mean (range)	12.3 (3 - 34)	16.2 (10 – 34)	7.2(3 – 10)	<0.001 ^1^

Values are mean ± SD, n (%) or n. AMH: Anti-Mullerian hormone, SLE: systemic lupus erythematosus. 1) t-test, 2) Chi-Square test.

The size of the follicles was 15-23 mm at the time of follicle aspiration. Oestradiol and LH concentrations level were significantly higher in the exposed group (Table I).

Premature ovulation occurred in 20.6% of women (95% confidence interval (CI) 9.2–30.6%) in the ibuprofen group and in 16.7% (4%-27.2%) of women in the control group, p=0.63. In total, 77 (85.5%) oocytes were obtained, 74 (96.1%) were Metaphase II and 59 (79.7%) were successfully fertilized. 88.1% of embryos developed to cleavage stage and were transferred. According to the ASEBIR assessment criteria embryos were classified as category A in 17 (32.7%), category B in 22 (42.3%), category C in 8 (15.4%) and category D in 5 (9.6%) cases. There was no difference in embryo quality between the exposed and the control group, Fisher’s exact test, p=0.360. In the exposed group it was category A: 10 (34.4%), B: 14 (48.3%), C: 4 (13.8%), D: 1 (3.5%) and in the control group it was category A: 7 (30.4%), B: 8 (34.8%), C: 4 (17.4%), D: 4 (17.4%). Cycles initiated because of male factor or idiopathic infertility revealed a better embryo quality compared to cycles initiated because of endometriosis. None of these outcome parameters differed significantly between the exposed and the control groups (Table II). In the regression analysis performed for increasing embryo quality none of the factors: age, reason for infertility, LH-level or ibuprofen exposure revealed any significant impact. The odds radio (OR) for each unit of LH (IU/l) and exposition to ibuprofen were above one (Table III).

**Table II:** Impact on female fertility. Comparison between ibuprofen exposed group and control group (no ibuprofen). Total number (N) and percentages per cycle, italic are the percentages per transfer.

	N=63	Ibuprofen exposed group	N=48	Control group	p-value
Premature ovulations	13	20.6 %	8	16.7 %	0.597 ^1^
Follicles aspirated	50	79.4 %	40	83.3 %	
Oocytes retrieved	41	65.1 %	36	75%	0.475 ^2^
Mature(MII) oocytes	39	61.9 %	35	72.9 %	0.223 ^2^
Oocytes fertilised per cycle	34	54 %	25	52 %	0.844 ^2^
Oocytes fertilised per MII		*87.2 %*		*71.4 %*	
Embryos transferred	29	46 %	23	47.9 %	0.844 ^2^
Number of implantations per cycle	8	12.7 %	3	6.3 %	0.116 ^2^
Number of implantations per transfer		*27.6%*		*13.0%*	
Deliveries per cycle	5	7.9 %	3	6.3	0.734 ^2^
Deliveries per transfer		*17.2%*		*13.0%*	

1) t-test, 2) Chi-Square test.

**Table III:** Regression analysis model for embryo quality (ASEBIR embryo assessment criteria).

	Odds Ratio	Std. Err.	p-value	[95% Conf. Interval]
Age 35y	Ref			
Age > 35y	0.87	0.46	0.79	0.31 2.44
				
LH-level (IU/l)	1.06	0.07	0.4	0.93 1.20
				
Idiopathic infertility (N=15)	Ref.			
Male factor infertility (N=26)	0.46	0.3	0.24	0.12 1.70
Tube factor infertility (N=2)	0.05	0.06	0.02	0.01 0.64
Endometriosis (N= 9)	0.67	0.52	0.6	0.15 3.06
				
Exposure to ibuprofen	1.8	1.45	0.47	0.37 8.76

LH-level as continuous, ibuprofen exposition as binary variable. y = years

Implantation and live birth rates were similar in both groups. 11 (21.1%) women’s embryos implanted successfully, leading to a clinical pregnancy and eight (15.4%) women delivered a healthy child. All children were born at term (mean 39.1 ± 1.0 weeks of gestation) with a normal birth weight (mean 3068 ± 345 g).

## Discussion

This is an observational study about the effect of the non-selective COX inhibitor ibuprofen on oocyte and embryo quality by analysing oocyte maturation, fertilisation rate, embryo development rate and embryo quality. Furthermore, implantation rate and delivery rates were calculated.

We present the first study testifying that ibuprofen does not have any influence on oocyte and embryo competence in NC-IVF. Because ibuprofen is effective and frequently used by fertile women in the treatment of pain conditions, such as headaches and dysmenorrhoea, it is important to understand the risks and mechanisms and its influence on oocyte competence and embryo quality. We do not assume a negative effect on oocyte and embryo parameters following the intake during early follicular (Uhler et al., 2001) or late pre-ovulatory phase intake, as shown by our results. However, whether this means that ibuprofen is completely harmless cannot be deducted from this data.

The strength of this study is the clearly defined study population and the high data quality. The NC-IVF treatment serves as a perfect model for the collection of this data, because there is no hormonal stimulation altering the process of oocyte maturation and embryo selection (Nargund et al., 2017; Datta et al., 2019; Von Wolff, 2019). Furthermore, NC-IVF allows the analysis of oocyte and embryo quality, which is not possible in spontaneous cycles. The data quality is very accurate because it is important for the fertility treatment of each woman. Accurate allocation and adherence to either the ibuprofen or the control group was checked by evaluating the blisters of ibuprofen at OPU.

The most striking limitation of the study is that the study patients were not randomised. Randomisation to analyse the impact of COX inhibitors without knowing if the study medication has a negative impact on fertility is hardly possible. Based on this study, which suggests that ibuprofen does not negatively affect the analysed fertility parameters; a prospective randomized study would be feasible.

A second limitation is that ibuprofen is given based on the LH-concentration at the time of ovulation triggering, leading to different LH- concentrations between the groups. In the regression analysis adjusted for LH level, no effect on embryo quality could be seen and this was independent of ibuprofen exposure. If LH had an effect, it would lead to differences in premature ovulation rates that were not different in both study groups.

A further limitation is the limited sample size regarding the analysis of implantation and delivery rates. These parameters were only secondary outcome criteria and due to the limited sample size, interpretation has to be performed with great care. The embryo transfer, clinical pregnancy and live birth rates correspond to previous studies about NC-IVF treatment (Von Wolff, 2019). With the limitation to only one potential oocyte per cycle, influences such as: preterm ovulation, the non- retrieval of the oocyte in the aspiration procedure and the lack of fertilisation or embryo development complicate the success of the therapy.

Non-selective COX inhibitors impede both COX I and COX II. COX I is found in many tissues responsible for functions such as platelet aggregation and gastric-acid secretion. COX II, referred to as the inducible form of cyclooxygenase, is expressed in selected cells involved in inflammation and is present in ovarian tissue. An association with cumulus expansion, oocyte release, follicle rupture and oocyte nuclear maturation has been postulated based on animal studies (Duffy, 2015). Selective COX II inhibitors, such as rofecoxib and meloxicam (Pall et al., 2001) have shown to completely block follicular lysis and oocyte detachment. In contrast, Killick and Elstein (1987) have shown that non- selective COX inhibitors such as indomethacin and azapropazone reduced ovulation rates, but did not completely block these PGE2 controlled processes. Based on our results, we can assume that ibuprofen acts in a similar manner.

Our findings on ibuprofen are in-line with sparse existing data, such as the prospective randomised crossover trial, where women took ibuprofen at a dosage of 800mg three times daily at the beginning of the cycle until the leading follicle reached 16mm. This study was designed to analyse the effect of ibuprofen on ovulation rates but not on oocyte and embryo competence. Matyas et al. (2015) analysed the effect of different kinds of COX inhibitors including ibuprofen taken in the early follicular but not the pre-ovulatory phase. They analysed the concentrations of reproductive hormones: Ibuprofen use during the follicular phase was associated with higher follicle stimulating hormone (FSH) and with lower oestradiol concentrations. The reproductive outcome was not studied. Other studies investigating non-selective COX inhibitors were either performed in animal models (Richards, 2001) or did not describe in what cycle phase the drug was applied at all (Matyas et al., 2015). According to the results of Matyas et al. (2015), the progesterone level during the luteal phase is not affected by ibuprofen. This seems to confirm that the hormonal cascade is still launched after the intake of ibuprofen and an effective ovulation favouring implantation occurs.

The effect of ibuprofen on the endometrium and its clinical relevance are still unclear. At the moment, there are only very few animal (St-Louis et al., 2010) and cell culture studies (Lundström et al., 1983; Seo et al., 2010) addressing this issue. It has been shown that celecoxib, a selective COX II inhibitor, increased “non-steroidal anti-inflammatory drug- activated gene-1” mRNA levels and consequently apoptosis in cultured human endometrial stromal cells (Seo et al., 2010). The short half-life of a non- selective COX-inhibitor, such as ibuprofen (90 minutes), might not be comparable to the effect of a long-acting COX II inhibitor, (for example celecoxib) with a half-life of 11 hours.

A small (N=10) study focusing on lysosomes in biopsied and cultured human endometrium following naproxen exposure does not suggest any functional impairment because of the non-selective COX inhibitor (Lundström et al., 1983).

Based on studies with indomethacin and azapropazone (Killick and Elstein, 1987; Priddy et al., 1990) both substances with a pharmacological half-life of six and twelve hours respectively, all COX inhibitors were not recommended for woman trying to conceive. Ibuprofen has a half-life of 90 minutes, so the short-term application of ibuprofen, even in the vulnerable pre-ovulatory phase does not affect oocyte competence or embryo quality.

Furthermore, in our study all the children were born at term, with normal birthweight and without any foetal malformations.

## Conclusions

Because oocyte maturation, embryo quality and implantation rates were not negatively affected by the intake of ibuprofen in our study, pre-ovulatory use of non-selective COX inhibitors seems to be without negative effects within NC-IVF. However, a randomised study with a larger sample size is needed to confirm this finding.
